# Evaluation of Anatomical and Tomographic Biomarkers as Predictive Visual Acuity Factors in Eyes with Retinal Vein Occlusion Treated with Dexamethasone Implant

**DOI:** 10.3390/jcm13154533

**Published:** 2024-08-02

**Authors:** Giuseppe Covello, Maria Novella Maglionico, Michele Figus, Chiara Busoni, Maria Sole Sartini, Marco Lupidi, Chiara Posarelli

**Affiliations:** 1 Department of Surgical, Medical, Molecular Pathology and Critical Care Medicine, University of Pisa, 56126 Pisa, Italy; m.novella.maglionico@gmail.com (M.N.M.); michele.figus@unipi.it (M.F.); chiarabuso@gmail.com (C.B.); chiara.posarelli@med.unipi.it (C.P.); 2 Ophthalmology, Department of Medical and Surgical Specialties, Azienda Ospedaliero Universitaria Pisana, 56124 Pisa, Italy; mssartini5@gmail.com; 3 Eye Clinic, Department of Experimental and Clinical Medicine, Polytechnic University of Marche, 60131 Ancona, Italy; marcomed2@gmail.com; 4 Fondazione per la Macula Onlus, Dipartimento di Neuroscienze, Riabilitazione, Oftalmologia, Genetica e Scienze Materno-Infantili (DINOGMI), University Eye Clinic, 16132 Genova, Italy

**Keywords:** retinal vein occlusion, macular edema, OCT, EZD, ELM, DRIL, MI

## Abstract

**Background:** This prospective study evaluated the impact of anatomical and tomographic biomarkers on clinical outcomes of intravitreal dexamethasone implants in patients with macular edema secondary to retinal vein occlusion (RVO). **Methods**: The study included 46 patients (28 with branch RVO (BRVO) and 18 with central RVO (CRVO)). Best corrected visual acuity (BCVA) significantly improved from a mean baseline of 0.817 ± 0.220 logMAR to 0.663 ± 0.267 logMAR at six months and 0.639 ± 0.321 logMAR at twelve months (*p* < 0.05). Central retinal thickness (CRT) showed a significant reduction from 666.2 ± 212.2 µm to 471.1 ± 215.6 µm at six months and 467 ± 175.7 µm at twelve months (*p* < 0.05). No significant differences were found in OCT biomarkers between baseline and follow-ups. **Results**: The study analysed improvements in visual acuity relative to baseline biomarkers. At six months, ellipsoid zone disruption (EZD) was significant for all subgroups. Disorganization of retinal inner layers (DRIL), external limiting membrane (ELM) disruption, macular ischemia (MI), CRT, and BRVO showed significance for any improvement, while DRIL and ELM were significant for changes greater than 0.3 logMAR (*p* < 0.05). At twelve months, EZD remained significant for all subgroups. ELM, MI, CRT, and BRVO were significant for any improvement, while MI and BRVO were significant for changes greater than 0.3 logMAR (*p* < 0.05). Hyperreflective foci were not statistically significant at either time point (*p* > 0.05). **Conclusions**: The regression model suggested that MI and CRVO could be negative predictive factors for visual outcomes, while ELM and EZD were associated with BCVA improvement one-year post-treatment.

## 1. Introduction

Retinal vein occlusion (RVO) is the second most common retinal vascular disease in the world affecting approximately 0.5% of people aged from 31 to 101 years [1,2]. According to anatomic location of occlusion, RVO can be divided into two main types: branch retinal vein occlusion (BRVO) and central retinal vein occlusion (CRVO). Visual loss is due to the development of macular edema (ME) and/or macular ischemia (MI). Blocked venous drainage induces the upregulation of vascular endothelial growth factor (VEGF) and inflammatory mediators, thereby increasing the permeability of vessels and causing a breakdown of the blood–retinal barrier. Treatment of macular edema is based on anti-VEGF intravitreal injections and steroid intravitreal implants. In addition to inhibiting the VEGF pathway, corticosteroids can also reduce the activity of other proinflammatory mediators [3]. The sustained-release intravitreal dexamethasone (DEX) implant, known as Ozurdex^®^ and manufactured by Allergan, Inc. in Irvine, CA, USA, was introduced as a treatment choice for patients with RVO-ME. The GENEVA study reported notably improved functional and anatomical outcomes in the eyes of RVO-ME patients following the administration of DEX [4,5]. The criteria to determine the appropriate intravitreal treatment are visual acuity (VA) and optical coherence tomography (OCT) parameters, such as type of edema (intraretinal, subretinal, cystoid) or central macular thickness. However, many studies have demonstrated a large variability in response to treatment protocols. After 1 year of anti-VEGF injections, although 47% to 70.7% of patients gain at least three VA lines, between 28.1% and 49.2% remain within three lines, and 1% to 5.9% lose at least three lines [6,7,8]. Beyond visual acuity, even OCT could show a reduction, a persistence, or an improvement in macular edema. Moreover, a correlation between VA and OCT parameters is not always present [9]. Despite resolution of macular edema, visual acuity does not always improve [10]. It is currently unclear how OCT imaging features relate to visual acuity in RVO-ME. Prior studies have investigated correlations between VA and some OCT parameters that have associations with biomarkers such as intraretinal hyperreflective foci (HRF); disorganization of the retinal inner layers (DRIL); the photoreceptor’s layers’ disruption such as external limiting membrane (ELM) and ellipsoid zone disruption (EZD); and retinal and choroidal thickness [11,12,13,14,15,16]. However, reaching definite conclusions is difficult because of the study design and sample size limitations, non-standardized treatment protocols, and failure to address other confounding variables (i.e., inner retinal changes, cysts, and cone outer segment tip visibility) [10,17,18]. For example, DRIL is an OCT feature represented by a disruption of any of the two boundaries between the ganglion cell inner plexiform layer, inner nuclear layer, and outer plexiform layer. In diabetic ME, reduced recovery of DRIL over 4 months after anti-VEGF treatment predicted VA worsening over 8 months, supporting a prognostic role for this marker [19,20]. Because ischemia in RVO impacts the inner retina, DRIL therefore may be a similarly useful biomarker [15]. The identification of OCT imaging characteristics that can predict clinical outcomes is essential for guiding the most effective treatment strategy. To establish correlations between anatomical and tomographic biomarkers and treatment results, the present study examined these parameters in a group of patients with RVO-ME who received DEX implant therapy.

## 2. Materials and Methods

This is a prospective, cohort study involving a population affected by RVO complicated by macular edema and treated with DEX implant between June 2022 and January 2023. This study was performed according to the tenets of the Declaration of Helsinki and approved by the Area Vasta Nord Ovest Ethical Committee (CEAVNO) with code number 22338. A written informed consent was signed by all the patients included in the study. Inclusion criteria comprised an angiographic diagnosis of RVO, both central or branch, after ophthalmoscopic evaluation, and the presence of RVO–ME, defined by a loss of the foveal pit and a central retinal thickness (CRT) > 250 micron on spectral domain optical coherence tomography (SD-OCT) [21]. Patients previously treated for RVO-ME or macular edema secondary to other any condition different to RVO (i.e., diabetes, uveitis, etc.), any intercurrent disease, either ophthalmic or systemic, that could prevent visual acuity recovery, or uncontrolled glaucoma (defined as a medically treated intraocular pressure > 24 mmHg) were excluded from the study. Moreover, images with poor quality due to eye movement, media opacities caused by corneal diseases and/or cataract, and thick retinal haemorrhages were excluded.

The OCT biomarkers were CRT, as explained above; DRIL; ELM; HRF; and EZD. All SD-OCT scans images were obtained using the Spectralis HRA + OCT2 platform (Heidelberg Engineering, Heidelberg, Germany) and conducted using a macular volumetric raster with dimensions of 30 × 25 degrees and 25-line scans spaced at 241 μm in high-speed (HS) mode with >12 automatic real-time tracking (ART). All biomarkers were evaluated in OCT scans passing through the fovea. CRT (µm) was calculated as the thickness of the central 1 mm circle in the ETDRS Grid. OCT biomarkers were measured by a single experienced ophthalmologist (G.C.). Particularly, DRIL, EZD, ELM and HRF were highlighted as binomial variables (present/absent).

The anatomical biomarkers were the type of RVO and the presence of macular ischemia using fluorescein angiography (FA) [21]. For FA evaluation, the ETDRS study grid was utilized, defining a non-perfusion area within the central 1 mm circle, with or without involvement of the inner and outer regions, as indicative of macular ischemia [22].

Patients were followed prospectively for 12 months and managed as in real life clinical practice in our institution. For each patient we collected data regarding best corrected visual acuity (BCVA) measured as logMAR; intraocular pressure (IOP) expressed as mmHg; the above-mentioned OCT and FA parameters; the number of injections; the number of visits and diagnostic procedures performed; and all the adverse events and the procedure related to them. Follow-up visits at six months and twelve months after the DEX injection were documented.

A 0.7 mg DEX implant (Ozurdex; Allergan, Inc.) was administered via intravitreal injection under aseptic conditions in an operating room. Two minutes before the injection, the patient’s study eye received an initial drop of oxibuprocaine (4 mg/mL), followed by a 5% povidone-iodine solution. The eyelid margins, eyelids, and periocular skin were cleansed with povidone-iodine. The eye was then draped in a sterile manner, and the surgeon inserted a sterile lid speculum. The implant was injected into the vitreous cavity using a 22-gauge needle. Patients were prescribed topical antibiotics, such as moxifloxacin, for seven days following the procedure.

In case of recurrent ME, a second DEX implant was injected after the six-month follow-up visit, adhering to the strict treatment regimen imposed by our region (Tuscany), which prohibits a second DEX implant within six months. Therefore, we scheduled follow-up visits at exactly six months to evaluate the necessity of additional treatment.

### 2.1. Outcomes

The primary endpoint was the mean change in BCVA from baseline to six- and twelve-month visits after treatment.

Secondary outcomes included the impact of the different OCT biomarkers on functional outcomes, expressed as BCVA at the two time points, and the impact of treatment on the different OCT biomarkers, reflected in their changes after the DEX implant at the two time points.

### 2.2. Statistical Analysis

SPSS IBM Corp. Released 2019 (IBM SPSS Statistics for Windows, Version 26.0. Armonk, NY, USA: IBM Corp) was used to perform the statistical analysis. Sample size was calculated a priori and indicated that 40 subjects were required, with a confidence level of 95% and a significance level of 5%, based on prior published studies [17,18,22]. Moreover, we performed a post hoc analysis to confirm the adequacy of the sample size [23]. The post hoc power analysis was conducted using an alpha level of 0.05, a power of 0.80, an effect size of 0.5 and the study sample size. After checking if any variable was normally distributed using the Shapiro–Wilk test, descriptive analyses were performed. Categorical variables were reported as counts and percentages and were compared with the chi-square test and Fisher’s exact test, as needed. To compare non-parametric values the Mann–Whitney U, Wilcoxon and McNemar tests were employed, as appropriate. A *p* value of less than 0.05 was considered significant. The ordinary least squares regression was used to understand the OCT biomarkers’ predictive value for visual acuity.

## 3. Results

Forty-six patients were enrolled after the evaluation of inclusion and exclusion criteria. Of those, 44 completed the 6-month follow-up visit and 40 patients completed the full study one year after the first DEX implant. Twenty-eight patients received a second implant due to the persistence of macular edema. Table 1 summarises the main demographic and clinical characteristics of the studied patients. Demographic characteristics showed that 24 (52.2%) patients were female and 22 (47.8%) were male. The mean age was 74.69 ± 12.49. Out of all the participants, 22 (47.8%) had undergone cataract surgery at the time of enrolment, and none of the patients developed a cataract severe enough to require surgical intervention.

As shown in Table 2, we observed a progressive, statistically significant improvement in visual acuity from a mean baseline value of 0.817 ± 0.220 logMAR to 0.663 ± 0.267 logMAR at six months and 0.639 ± 0.321 logMAR at twelve months (*p* < 0.05). There has been a statistically significant decrease in CRT at both follow-up visits, with the mean value declining from 666.17 ± 212.21 µm at the baseline visit to 471.15 ± 215.63 µm after six months and further to 467 ± 175.67 µm at the twelve-month visit. Any statistically significant differences emerged between baseline and follow-up visits regarding OCT biomarkers.

Functional outcomes were categorized into two subgroups: those showing any improvement in BCVA and those with a ≤ 0.3 logMAR improvement. This division aimed to provide a more detailed analysis of the variables and to gain insights into how biomarkers affect visual acuity over time. In Table 3, comparisons between improvements in visual acuity and OCT biomarkers were presented, complete with 95% confidence intervals (CIs) and odds ratios (ORs) for significant values. In this analysis, anatomical biomarkers were considered, with macular ischemia classified as absent, and the anatomical variant of BRVO selected for evaluation. At the six-month visit, EZD exhibited significance for all subgroups. Furthermore, DRIL, ELM, macular ischemia, CRT, and BRVO showed significance for any improvement, while for changes of more than 0.3 logMAR, statistical significance was observed for DRIL and ELM only (*p* < 0.05).

However, at twelve months, EZD remained significant for all subgroups. ELM, macular ischemia, CRT, and BRVO displayed significance for any improvement, while for changes of more than 0.3 logMAR, statistical significance was observed for MI and BRVO (*p* < 0.05). Notably, HRF did not exhibit statistical significance at either time point (*p* > 0.05).

**Figure 1:**
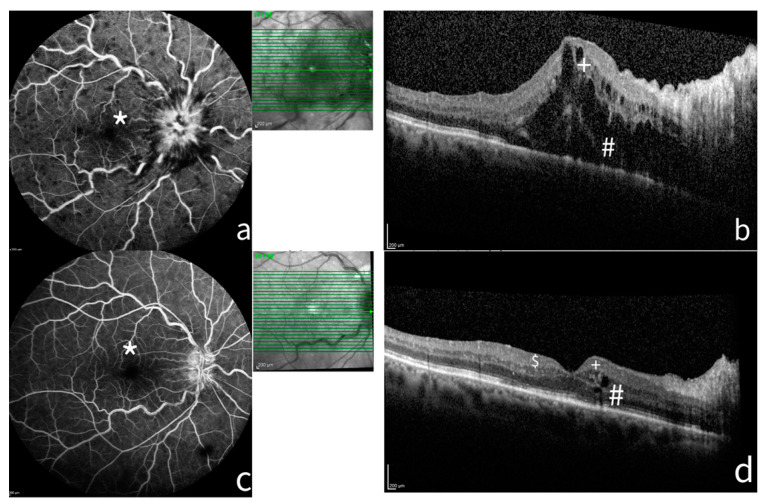
A case of CRVO at baseline and six months after treatment. FA and SD-OCT of a patient before and after treatment. (**a**) FA shows a non-ischemic (*) central retinal vein occlusion (CRVO) with marked delay in arteriovenous transit time, masked by retinal haemorrhages, and vessel wall staining. (**b**) The SD-OCT shows ME with considerable central thickness, absence of DRIL (+), and incomplete disruption of ELM and EZ. (**c**) After six months of treatment, FA shows resolution of the RVO with late staining of optic disc. (**d**) The SD-OCT highlights the reduction in retinal thickness, the presence of HRF ($), resolution of photoreceptor layers (#), and absence of DRIL (+). FA: Fluorescein angiograms; SD-OCT: spectral domain–optical coherence tomography; ME: macula edema; DRIL: disorganization of retinal inner layers; ELM: external limiting membrane disruption; HRF: hyperreflective foci; EZD: ellipsoid zone disruption.

Disruptions of photoreceptors’ layers showed a significant association with visual outcome as well as the absence of macular ischemia at baseline and the branch type of RVO at twelve months (*p* < 0.05). Figure 1 shows a case of CRVO at baseline and at six months after treatment.

### 3.1. Role of Macular Ischemia

As indicated by the prior analysis, macular ischemia emerges as a potential negative predictive factor for visual recovery. 

To delve deeper into the role of OCT biomarkers in predicting visual outcomes in patients with baseline macular ischemia, we focused on patients presenting with MI (n = 28). We then conducted a comparative analysis of their baseline biomarkers in relation to BCVA at 6 and 12 months, using the chi-squared test. The findings, detailed in Table 4, revealed that the OCT biomarkers, specifically ELM and EZD, exhibited statistical significance concerning visual recovery both at the 6- and 12-month visits (*p* < 0.05 for both). Additionally, it is worth noting that BRVO appeared to be a prospective positive predictive factor at both time points, especially regarding improvements exceeding 0.3 logMAR.

### 3.2. Predictive Factors of Visual Acuity

To identify potential predictors of improved visual acuity at 6 and 12 months, we ran the ordinary least squares regression (R-squared: 0.697; coeff.: 0.4446; std err.: 0.207; t: 2.147; P > |t|:0.040; CI: 0.022 to 0.867). All the baseline biomarkers, both anatomical and tomographic, were considered as variables. At 6 months, none of the variables showed a statistical significance (*p* > 0.05). At the final visit, baseline macular ischemia (coeff.: −0.2733; *p*: 0.009; CI: −0.472 to −0.075) and the branch type of RVO (coeff.: 0.3506; *p*: 0.005; CI: 0.111 to 0.590) were retained as predictive factors for final BCVA. 

Twenty-eight patients (60.8%) required rescue therapy. Ten patients were affected by CRVO and eighteen by BRVO, without statistical differences (*p*: 0.76; OR: 0.69, 95% CI: 0.21 to 2.33). 

Safety analysis revealed no major complications such as endophthalmitis or insert dislocation to the anterior chamber. No patient showed signs of glaucoma before the DEX implant. Moreover, no patient experienced a significant increase (≥5 mmHg) in IOP or other adverse events.

## 4. Discussion

The results of this study confirmed the DEX implant’s effectiveness in significantly improving visual acuity in patients affected by RVO-ME. BCVA improved from a baseline mean value of 0.817 ± 0.220 logMAR to 0.663 ± 0.267 logMAR at six months and 0.639 ± 0.321 logMAR at twelve months. Meanwhile, the DEX implant significantly reduced CRT from a mean baseline value of 666.17 ± 212.21 µm to 471.15 ± 215.63 µm and 467 ± 175.67 µm.

The functional and anatomical improvements observed in our study were consistent with existing scientific evidence [16,24,25]. Furthermore, the DEX implant appeared to contribute to the restoration of retinal status (DRIL, ELM, EZD, HRF), albeit without reaching statistical significance. This finding aligns with a prior study conducted by Castro-Navarro et al. [16], which reported that the DEX implant significantly improved ELM integrity in patients with macular edema secondary to retinal vascular disease, although a subsequent study failed to replicate these findings [22]. The authors attributed this inconsistency to differences in the patient sample, with the first study encompassing both diabetic and RVO patients. Additionally, the evaluation of ELM changes in the first study was quantitative, while the second employed a qualitative approach. Our study shared the same limitation: OCT biomarkers were considered as binomial variables (absent/present). However, some OCT biomarkers such as ELM, EZD, and CRT have shown a statistical association with visual improvement at either 6 or 12 months, whereas DRIL only at six months. No significant associations were observed in DRIL at 12 months and HRF. Despite these associations, the ordinary least squares regression results did not observe any relationship between all baseline OCT biomarkers and the clinical outcomes. These results are consistent with previous evidence in the literature, which also emphasized the lack of uniform findings among different studies [15,22,26]. It is possible that a quantitative analysis may have yielded more informative results on these biomarkers. For instance, it has been observed that a higher HRF count at baseline was associated with improved visual outcomes following anti-VEGF injections as a reduction in HRF was correlated with a visual acuity improvement after DEX implant [27,28]. Instead, in our study, HRF did not seem to have any relationship with visual outcomes. Moreover, the definition of HRF in RVO remains controversial. Notably, two distinct HRF populations were identified: fine scattered HRF, likely associated with the leakage of blood constituents, and confluent HRF, mainly found in unaffected areas spared by the retinal occlusion [12,29]. Confluent HRF are believed to be linked to the absorption of water and other molecules. While fine scattered HRF are not visible on fundus photographic images, confluent HRF are thought to represent retinal exudates. In RVO, HRF are distributed topographically along the outer plexiform layer and the ELM. Like other retinal diseases, the presence of HRF at baseline is associated with poor visual outcomes following anti-VEGF treatment. Additionally, DEX implants might be preferred for eyes with numerous HRF and long-standing macular edema secondary to RVO, due to the inflammatory component [29]. However, likely due to the limited sample size, we did not find significant associations between changes in BCVA and HRF. Therefore, further studies are needed to understand the exact definition and role of HRF to establish their diagnostic significance.

Fluorescein angiography remains the gold standard of care imaging modality in determining the risk of neovascularization and areas of retinal non-perfusion after RVOs. The detection of non-perfused retinal areas by standard FA is the basis for the classification of RVOs as “ischemic” or “non-ischemic” [30]. In case of absence of neovascularization, vitreous haemorrhage, tractional retinal detachment or neovascular glaucoma, visual loss is related to macular edema or macular ischemia. Macular edema is well diagnosed and quantified by OCT and treated with anti-VEGF injections or corticosteroids. Nonetheless, the visual prognosis following treatment often hinges on the existence and extent of macular ischemia, along with photoreceptor loss or atrophy. Macular ischemia can be confirmed with FA and OCT angiography (OCTA) [21]. In OCTA, this is seen as an enlarged, irregular foveal avascular zone (FAZ). Some studies have found significant correlations between visual acuity and OCTA parameters such as FAZ, suggesting that OCTA metrics can be useful biomarkers for identifying and monitoring macular ischemia, and can be informative for visual prognoses in RVOs [31,32]. However, in this study, macular ischemia was investigated with fluorescein angiography. This approach was chosen for its effectiveness in both eliminating potential differential diagnoses and assessing the presence of macular ischemia as well as large areas of non-perfusion [33].

Our results have confirmed a significant association between macular ischemia and visual acuity. Patients without MI exhibited greater visual improvement compared to those with MI. Furthermore, the MI biomarker has emerged as a negative predictive factor for visual acuity. To gain a deeper understanding of MI’s role, we analysed patients who presented with MI at baseline and explored the associations between OCT biomarkers and visual outcomes.

In this analysis, we found that the integrity of two specific OCT biomarkers, ELM and EZD, demonstrated statistically significant associations (*p* < 0.05). These results align with previous studies [16,33,34] that established a relationship between photoreceptor’s integrity and visual improvement. Etheridge et al. [35] suggested that the early recovery of the EZ may be a crucial driver of visual outcomes in patients with RVO. The prompt improvement in EZ integrity likely signifies the resolution of macular edema, which can disrupt photoreceptors. This leads to the re-approximation and/or organization of this visually essential retinal layer. Liu et al. [36] found that an intact initial ELM predicts better visual outcomes after anti-VEGF treatment in RVO patients.

Taken together, these findings suggest that the presence of macular ischemia may limit the restoration of these retinal layers despite treatment. To date, no consensus exists on the extent or location of macular non-perfusion on FA or OCT angiography that can cause loss of vision [21]. FA can more effectively recognize the extent of capillary non-perfusion (CNP) in both the peripheral retina and macula by visualizing areas of the retina with capillary dropout. However, the true severity of CNP, which correlates with the overall metabolic changes resulting from retinal ischemia and ultimately influencing the anatomical and functional outcomes, cannot be comprehensively evaluated through angiography alone [37]. In fact, the interaction of various biochemical factors may contribute to the final clinical presentation, and many of these factors still escape our complete understanding. Therefore, we should explore approaches beyond angiographic assessment to achieve a more accurate evaluation of CNP severity. In this regard, some promising studies have been published concerning the measurement of oxygen saturation and blood flow in cases of retinal vein occlusions [38,39]. 

It is worth noting that further research is needed, particularly in the form of histologic investigations, to provide a more comprehensive understanding of these relationships.

Meanwhile, the type of RVO, particularly CRVO, has shown a negative predictive factor for visual acuity. This might be attributed to the continuous involvement of the macular region in CRVO-affected patients, whereas BRVO may only partially affect the macular region. This highlights the crucial role of macular, especially foveal, region integrity in visual prognosis [31,33].

This study presents some strengths and limitations. The strengths are as follows: (i) the bias resulting from the type of agent was controlled; the DEX implant was the single intravitreal agent; (ii) and the prospective nature of this protocol with well-defined inclusion/exclusion criteria, strict treatment regimen and follow-up schedule, and standardized protocols for SD-OCT imaging and visual acuity measurements. This study was limited by a small sample size and the presentation of tomographic biomarkers as binomial variables. Measurements and quantitative representation of data as well as a greater sample could change the analysis and the interpretation of results. The automation of OCT measurements using OCT softwares may offer utility in future treatment approaches and in the assessment of visual acuity, as already seen for diabetic macular edema [40]. Moreover, this study did not incorporate other imaging modalities such as OCT angiography (OCT-A). Future studies employing multimodal imaging may improve the predictive power of SD-OCT biomarkers.

## 5. Conclusions

The DEX implant was confirmed to be an effective treatment in eyes with RVO-ME, on both visual acuity and CRT. The results of this study suggest that DEX is a good influence when restoring tomographic biomarkers. Macular ischemia and CRVO were negative predictive factors for visual outcomes, whereas external limiting membrane and ellipsoid zone disruptions revealed a statistical association with BCVA improvement one year after treatment. Anatomical and tomographic biomarkers are confirmed to be useful to predict treatment response and monitor disease progression. Emerging OCT technologies have demonstrated innovative imaging biomarkers that hold the potential to refine the stratification of treatment responses and aid in medical management choices. These innovative markers complement traditional diagnostic methods like fluorescein angiography, which continue to play a pivotal role in confirming diagnoses and guiding medical decisions. Further studies are needed to better elucidate the impact and predictivity of these and other potential biomarkers.

## Figures and Tables

**Table 1:** Demographic and clinical data of patients.

	Baseline (n = 46)
Age (years) mean ± SD 95% CI	74.69 ± 12.49 68.92 to 77.28
Gender, female n (%)	24 (52.2%)
Gender, male n (%)	22 (47.8%)
Right eye n (%)	22 (47.8%)
Left eye n (%)	24 (52.2%)
Type-BRVO n (%)	28 (60.8%)
Type-CRVO n (%)	18 (39.2%)
Macular ischemia, n (%)	28 (60.8%)
Pseudophakia, n (%)	22 (47.8%)
Second implant, n (%)	28 (60.8%)
IOP, mmHg ± SD, 95% CI	16.4 ± 1.4 15.9 to 16.8
BCVA, mean ± SD, 95% CI	0.817 ± 0.220 0.4 to 1
CRT, µm ± SD, 95% CI	666.2 ± 212.2 596.1 to 735.7

N: number; SD: standard deviation; CI: confidence interval: BRVO: branch retinal vein occlusion; CRVO: central retinal vein occlusion, IOP: intraocular pressure.

**Table 2:** Functional and tomographic parameters at each follow-up visit.

	Baseline (n = 46)	6 Months (n = 44)		12 Months (n = 40)	
BCVA, mean ± SD, 95% CI	0.817 ± 0.220 0.4 to 1	0.663 ± 0.267 0.3 to 1	***p* < 0.05** ^a^	0.639 ± 0.321 0.2 to 1	***p* < 0.05** ^a^
CRT, µm ± SD 95% CI	666.2 ± 212.2 596.1 to 735.7	471.1 ± 215.6 399.8 to 542.5	***p* < 0.05** ^a^	467 ± 175.7 409.9 to 525.4	***p* < 0.05** ^a^
DRIL (yes %)	65.2%	60.9%	*p* = 0.5 ^b^	56.5%	*p* = 0.3 ^b^
ELM (yes %)	47.8%	47.8%	*p* > 0.9 ^b^	43.5%	*p* = 0.7 ^b^
HRF (yes %)	69.6%	78.3%	*p* = 0.3 ^b^	56.5%	*p* = 0.1 ^b^
EZD (yes %)	52.2%	47.8%	*p* = 0.7 ^b^	47.8%	*p* = 0.7 ^b^

N: number; BCVA: best corrected visual acuity; CRT: central retinal thickness; DRIL: disorganization of retinal inner layers; ELM: external limiting membrane disruption; HRF: hyperreflective foci; EZD: ellipsoid zone disruption; ^a^: Wilcoxon test; ^b^: McNemar test. Bold character is for statistically significant values.

**Table 3:** Comparisons between baseline biomarkers and BCVA at 6 and 12 months using chi-squared test (*p* < 0.05 was considered for significance).

Baseline Anatomic and OCT Biomarkers	*p* for Any BCVA Improvement at 6 Months	*p* for Improved BCVA ≤ 0.3 at 6 Months	*p* for Any BCVA Improvement at 12 Months	*p* for Improved BCVA ≤ 0.3 at 12 Months
DRIL OR, 95% CI	**0.021** 0.14, 0.03 to 0.76	**0.008** 0.13, 0.03 to 0.59	0.143	0.127
ELM OR, 95% CI	**<0.001** 0.04, 0.01 to 0.02	**0.032** 0.17, 0.06 to 0.80	**<0.001** 0.32, 0.01 to 0.2	0.101
HRF OR, 95% CI	0.506	0.739	0.698	0.684
EZD OR, 95% CI	**<0.001** 0.05, 0.01 to 0.3	**0.012** 0.07, 0.03 to 0.64	**<0.001** 0.05, 0.01 to 0.31	**0.042** 0.2, 0.5 to 0.84
MI OR, 95% CI	**0.005** 0.1, 0.02 to 0.56	**0.027** 0.22, 0.06 to 0.79	**0.002** 0.07, 0.11 to 0.41	**0.002** 0.07, 0.11 to 0.41
CRT	**0.006** ^a^	0.854 ^a^	**0.012** ^a^	0.191 ^a^
BRVO OR, 95% CI	0.054	0.210	**0.008** 0.13, 0.03 to 0.59	**<0.001** 0.42, 0.26 to 0.67

^a^: Mann–Whitney U test; OCT: optical coherence tomography; BCVA: best corrected visual acuity; CRT: central retinal thickness; DRIL: disorganization of retinal inner layers; ELM: external limiting membrane disruption; HRF: hyperreflective foci; EZD: ellipsoid zone disruption; MI: macular ischemia; BRVO: branch retinal vein occlusion; OR: odds ratio. Bold character is for statistically significant values.

**Table 4:** Comparisons between biomarkers and visual improvement in 28 patients with macular ischemia.

Baseline Anatomic and OCT Biomarkers	*p* Value for Any BCVA Improvement at 6 Months	*p* Value for Improved BCVA ≤ 0.3 at 6 Months	*p* Value for Any BCVA Improvement at 12 Months	*p* Value for Improved BCVA ≤ 0.3 at 12 Months
DRIL OR, 95% CI	0.492	>0.99	0.492	>0.99
ELM OR, 95% CI	**<0.001** 0.03, 0.003 to 0.24	0.354	**<0.001** 0.2, 0.03 to 0.2	0.354
HRF OR, 95% CI	0.673	0.289	0.673	0.289
EZD OR, 95% CI	**0.005** 0.07, 0.01 to 0.48	0.147	**0.005** 0.07, 0.01 to 0.48	0.147
BRVO OR, 95% CI	**0.023** 0.12, 0.02 to 0.74	**0.024** 0.62, 0.43 to 0.91	**0.023** 0.12, 0.19 to 0.74	**0.024** 0.62, 0.4 to 0.9

OCT: optical coherence tomography; BCVA: best corrected visual acuity; DRIL: disorganization of retinal inner layers; ELM: external limiting membrane disruption; HRF: hyperreflective foci; EZD: ellipsoid zone disruption; BRVO: branch retinal vein occlusion; OR: odds ratio. Bold character is for statistically significant values.

## Data Availability

Data are fully available upon specific and motivated request to the authors.

## References

[1] Wong T.Y., Scott I.U. (2010). Retinal-Vein Occlusion. N. Engl. J. Med..

[2] Rogers S., McIntosh R.L., Cheung N., Lim L., Wang J.J., Mitchell P., Kowalski J.W., Nguyen H., Wong T.Y. (2010). The Prevalence of Retinal Vein Occlusion: Pooled Data from Population Studies from the United States, Europe, Asia, and Australia. Ophthalmology.

[3] Sohn H.J., Han D.H., Kim I.T., Oh I.K., Kim K.H., Lee D.Y., Nam D.H. (2011). Changes in Aqueous Concentrations of Various Cytokines After Intravitreal Triamcinolone Versus Bevacizumab for Diabetic Macular Edema. Arch. Ophthalmol..

[4] Haller J.A., Bandello F., Belfort R., Blumenkranz M.S., Gillies M., Heier J., Loewenstein A., Yoon Y.-H., Jacques M.-L., Jiao J. (2010). Randomized, Sham-Controlled Trial of Dexamethasone Intravitreal Implant in Patients with Macular Edema Due to Retinal Vein Occlusion. Ophthalmology.

[5] Haller J.A., Bandello F., Belfort R., Blumenkranz M.S., Gillies M., Heier J., Loewenstein A., Yoon Y.H., Jiao J., Li X.-Y. (2011). Dexamethasone Intravitreal Implant in Patients with Macular Edema Related to Branch or Central Retinal Vein Occlusion. Ophthalmology.

[6] Korobelnik J.F., Holz F.G., Roider J., Ogura Y., Simader C., Schmidt-Erfurth U., Lorenz K., Honda M., Vitti R., Berliner A.J. (2014). Intravitreal Aflibercept Injection for Macular Edema Resulting from Central Retinal Vein Occlusion. Ophthalmology.

[7] Brown D.M., Heier J.S., Clark W.L., Boyer D.S., Vitti R., Berliner A.J., Zeitz O., Sandbrink R., Zhu X., Haller J.A. (2013). Intravitreal Aflibercept Injection for Macular Edema Secondary to Central Retinal Vein Occlusion: 1-Year Results From the Phase 3 COPERNICUS Study. Arch. Ophthalmol..

[8] Larsen M., Waldstein S.M., Boscia F., Gerding H., Monés J., Tadayoni R., Priglinger S., Wenzel A., Barnes E., Pilz S. (2016). Individualized Ranibizumab Regimen Driven by Stabilization Criteria for Central Retinal Vein Occlusion. Ophthalmology.

[9] Ota M., Tsujikawa A., Kita M., Miyamoto K., Sakamoto A., Yamaike N., Kotera Y., Yoshimura N. (2008). Integrity of Foveal Photoreceptor Layer in Central Retinal Vein Occlusion. Retina.

[10] Ko J., Kwon O.W., Byeon S.H. (2014). Optical Coherence Tomography Predicts Visual Outcome in Acute Central Retinal Vein Occlusion. Retina.

[11] Jonas J.B., Monés J., Glacet-Bernard A., Coscas G. (2017). Retinal Vein Occlusions. Macular Edema.

[12] Bin M., Hai-Ying Z., Xuan J., Feng Z. (2017). Evaluation of hyperreflective foci as a prognostic factor of visual outcome in retinal vein occlusion. Int. J. Ophthalmol..

[13] Moon B.G., Cho A.R., Kim Y.N., Kim J.G. (2018). Predictors of Refractory Macular Edema After Branch Retinal Vein Occlusion Following Intravitreal Bevacizumab. Retina.

[14] Banaee T., Singh R.P., Champ K., Conti F.F., Wai K., Bena J., Beven L., Ehlers J.P. (2018). Ellipsoid Zone Mapping Parameters in Retinal Venous Occlusive Disease with Associated Macular Edema. Ophthalmol. Retin..

[15] Babiuch A.S., Han M., Conti F.F., Wai K., Silva F.Q., Singh R.P. (2019). Association of Disorganization of Retinal Inner Layers with Visual Acuity Response to Anti–Vascular Endothelial Growth Factor Therapy for Macular Edema Secondary to Retinal Vein Occlusion. JAMA Ophthalmol..

[16] Castro-Navarro V., Monferrer-Adsuara C., Navarro-Palop C., Montero-Hernández J., Cervera-Taulet E. (2021). Effect of Dexamethasone Intravitreal Implant on Visual Acuity and Foveal Photoreceptor Integrity in Macular Edema Secondary to Retinal Vascular Disease. Ophthalmologica.

[17] Shin H.J., Chung H., Kim H.C. (2011). Association between integrity of foveal photoreceptor layer and visual outcome in retinal vein occlusion. Acta Ophthalmol..

[18] Mitamura Y., Fujihara-Mino A., Inomoto N., Sano H., Akaiwa K., Semba K. (2016). Optical coherence tomography parameters predictive of visual outcome after anti-VEGF therapy for retinal vein occlusion. Clin. Ophthalmol..

[19] Radwan S.H., Soliman A.Z., Tokarev J., Zhang L., van Kuijk F.J., Koozekanani D.D. (2015). Association of Disorganization of Retinal Inner Layers With Vision After Resolution of Center-Involved Diabetic Macular Edema. JAMA Ophthalmol..

[20] Sun J.K., Lin M.M., Lammer J., Prager S., Sarangi R., Silva P.S., Aiello L.P. (2014). Disorganization of the Retinal Inner Layers as a Predictor of Visual Acuity in Eyes With Center-Involved Diabetic Macular Edema. JAMA Ophthalmol..

[21] Schmidt-Erfurth U., Garcia-Arumi J., Gerendas B.S., Midena E., Sivaprasad S., Tadayoni R., Wolf S., Loewenstein A. (2019). Guidelines for the Management of Retinal Vein Occlusion by the European Society of Retina Specialists (EURETINA). Ophthalmologica.

[22] Castro-Navarro V., Monferrer-Adsuara C., Navarro-Palop C., Montero-Hernández J., Cervera-Taulet E. (2022). Optical coherence tomography biomarkers in patients with macular edema secondary to retinal vein occlusion treated with dexamethasone implant. BMC Ophthalmol..

[23] Phadnis M.A. (2019). Sample size calculation for small sample single-arm trials for time-to-event data: Logrank test with normal approximation or test statistic based on exact chi-square distribution?. Contemp. Clin. Trials Commun..

[24] Ji K., Zhang Q., Tian M., Xing Y. (2019). Comparison of dexamethasone intravitreal implant with intravitreal anti-VEGF injections for the treatment of macular edema secondary to branch retinal vein occlusion. Medicine.

[25] Li X., Wang N., Liang X., Xu G., Li X.-Y., Jiao J., Lou J., Hashad Y., China Ozurdex in RVO Study Group (2018). Safety and efficacy of dexamethasone intravitreal implant for treatment of macular edema secondary to retinal vein occlusion in Chinese patients: Randomized, sham-controlled, multicenter study. Graefe’s Arch. Clin. Exp. Ophthalmol..

[26] Midena E., Torresin T., Schiavon S., Danieli L., Polo C., Pilotto E., Midena G., Frizziero L. (2023). The Disorganization of Retinal Inner Layers Is Correlated to Müller Cells Impairment in Diabetic Macular Edema: An Imaging and Omics Study. Int. J. Mol. Sci..

[27] Luís M.E., Sampaio F., Costa J., Cabral D., Teixeira C., Ferreira J.T. (2021). Dril Influences Short-term Visual Outcome after Intravitreal Corticosteroid Injection for Refractory Diabetic Macular Edema. Curr. Eye Res..

[28] American Diabetes Association (2013). Diagnosis and Classification of Diabetes Mellitus. Diabetes Care.

[29] Fragiotta S., Abdolrahimzadeh S., Dolz-Marco R., Sakurada Y., Gal-Or O., Scuderi G. (2021). Significance of Hyperreflective Foci as an Optical Coherence Tomography Biomarker in Retinal Diseases: Characterization and Clinical Implications. J. Ophthalmol..

[30] Tan T.-E., Ibrahim F., Chandrasekaran P.R., Teo K.Y.C. (2023). Clinical utility of ultra-widefield fluorescein angiography and optical coherence tomography angiography for retinal vein occlusions. Front. Med..

[31] Wons J., Pfau M., Wirth M.A., Freiberg F.J., Becker M.D., Michels S. (2016). Optical Coherence Tomography Angiography of the Foveal Avascular Zone in Retinal Vein Occlusion. Ophthalmologica.

[32] Salles M.C., Kvanta A., Amrén U., Epstein D. (2016). Optical Coherence Tomography Angiography in Central Retinal Vein Occlusion: Correlation Between the Foveal Avascular Zone and Visual Acuity. Investig. Ophthalmol. Vis. Sci..

[33] Antropoli A., Bianco L., Arrigo A., Bandello F., Parodi M.B. (2023). Non-perfusion severity correlates with central macular thickness and microvascular impairment in branch retinal vein occlusions. Eur. J. Ophthalmol..

[34] De S., Saxena S., Kaur A., Mahdi A.A., Misra A., Singh M., Meyer C.H., Akduman L. (2021). Sequential restoration of external limiting membrane and ellipsoid zone after intravitreal anti-VEGF therapy in diabetic macular oedema. Eye.

[35] Etheridge T., Dobson E.T.A., Wiedenmann M., Oden N., VanVeldhuisen P., Scott I.U., Ip M.S., Eliceiri K.W., Blodi B.A., Domalpally A. (2021). Ellipsoid Zone Defects in Retinal Vein Occlusion Correlates with Visual Acuity Prognosis: SCORE2 Report 14. Transl. Vis. Sci. Technol..

[36] Liu H., Li S., Zhang Z., Shen J. (2017). Predicting the visual acuity for retinal vein occlusion after ranibizumab therapy with an original ranking for macular microstructure. Exp. Ther. Med..

[37] Parodi M.B., Arrigo A., Antropoli A., Bianco L., Saladino A., Bandello F., Vilela M., Mansour A. (2023). Deep Capillary Plexus as Biomarker of Peripheral Capillary Nonperfusion in Central Retinal Vein Occlusion. Ophthalmol. Sci..

[38] Šínová I., Řehák J., Nekolová J., Jirásková N., Haluzová P., Řeháková T., Bábková B., Hejsek L., Šín M. (2018). Correlation Between Ischemic Index of Retinal Vein Occlusion and Oxygen Saturation in Retinal Vessels. Arch. Ophthalmol..

[39] Nicholson L., Vazquez-Alfageme C., Hykin P.G., Bainbridge J.W., Sivaprasad S. (2019). The Relationship Between Retinal Vessel Oxygenation and Spatial Distribution of Retinal Nonperfusion in Retinal Vascular Diseases. Investig. Ophthalmol. Vis. Sci..

[40] Midena E., Toto L., Frizziero L., Covello G., Torresin T., Midena G., Danieli L., Pilotto E., Figus M., Mariotti C. (2023). Validation of an Automated Artificial Intelligence Algorithm for the Quantification of Major OCT Parameters in Diabetic Macular Edema. J. Clin. Med..

